# The association between T wave inversion in leads with ST-elevation and patency of the infarct-related artery

**DOI:** 10.1186/s12872-021-01851-8

**Published:** 2021-01-12

**Authors:** Abdolmohammad Ranjbar, Bahram Sohrabi, Seyyed-Reza Sadat-Ebrahimi, Samad Ghaffari, Babak Kazemi, Naser Aslanabadi, Babak Seyvani, Reza Hajizadeh

**Affiliations:** 1 Cardiovascular Research Center, Tabriz University of Medical Science, Tabriz, Iran; 2 Department of Cardiology, Urmia University of Medical Sciences, Urmia, Iran

**Keywords:** ST-elevation myocardial infarction, T wave inversion, Patency, Infarct related artery

## Abstract

**Background:**

Up to over half of the patients with ST-segment elevation myocardial infarction (STEMI) are reported to undergo spontaneous reperfusion without therapeutic interventions. Our objective was to evaluate the applicability of T wave inversion in electrocardiography (ECG) of patients with STEMI as an indicator of early spontaneous reperfusion.

**Methods:**

In this prospective study, patients with STEMI admitted to a tertiary referral hospital were studied over a 3-year period. ECG was obtained at the time of admission and patients underwent a PPCI. The association between early T wave inversion and patency of the infarct-related artery was investigated in both anterior and non-anterior STEMI.

**Results:**

Overall, 1025 patients were included in the study. Anterior STEMI was seen in 592 patients (57.7%) and non-anterior STEMI in 433 patients (42.2%). Among those with anterior STEMI, 62 patients (10.4%) had inverted T and 530 (89.6%) had positive T waves. In patients with anterior STEMI and inverted T waves, a significantly higher TIMI flow was detected (*p *value = 0.001); however, this relationship was not seen in non-anterior STEMI.

**Conclusion:**

In on-admission ECG of patients with anterior STEMI, concomitant inverted T wave in leads with ST elevation could be a proper marker of spontaneous reperfusion of infarct related artery.

## Background

Primary percutaneous coronary intervention (PPCI) and thrombolytic therapy have been suggested as the essential therapeutic techniques in the management of patients with acute ST-elevation myocardial infarction (STEMI) [1]. It has been reported by some angiographic studies that 7–57% of patients with STEMI developed spontaneous reperfusion (SR) prior to PPCI [2–7]. In such cases, fibrinolytic therapy may not be advantageous in salvaging the myocardial ischemia because the culprit vessel is already partially patent and fibrinolytic therapy may enhance bleeding risk. Therefore, initial conservative treatment for patients with SR has been proposed and supported as a safe strategy by some previous investigations [4, 5, 8]. However, current guidelines of AHA/ACC and ESC do not consider spontaneous reperfusion as a contraindication of PPCI or thrombolytics in patients with STEMI [1, 9]. Due to the time limitations of cardiac interventions for STEMI patients, imaging studies or other laboratory tests could not be considered prior to PPCI for evaluation of presence of SR in infarct-related artery (IRA). However, electrocardiogram (ECG) can be used as an available, rapid, and easily interpretable tool in these situations. Atar et al. have described the ECG markers of reperfusion including resolution of ST-segment elevation, altered QRS appearance, T wave changes, and reperfusion arrhythmias [10]. A few studies have demonstrated that early T wave inversion can be regarded as a useful marker indicating spontaneous subendocardial reperfusion [8, 11, 12]. It was also associated with a higher patency rate of IRA and improvement in left ventricular function [11, 12]. However, the associations between T wave inversion and angiographic findings have not yet been evaluated in large-scale studies. Although ST elevation resolution more than 50% is a good marker for reperfusion in patients with at least two serial ECGs, it could not be used in patients with single ECG, presenting to emergency room with relived chest pain and 1–2 mm ST elevation and no base ECG. The aim of this study was to evaluate the association of on admission T wave inversion in the presenting ECG of acute STEMI patients undergoing PPCI with spontaneous reperfusion of the infarct related artery.

## Methods

**Fig. 1:**
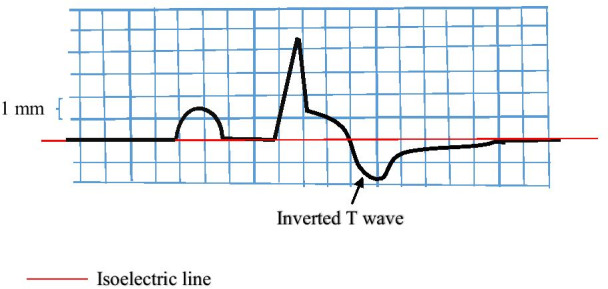
The method used for defining an inverted T wave in ST-elevation myocardial infarction

In this prospective study, patients with acute STEMI undergoing PPCI from May 2016 to May 2019 were evaluated. Patients with a history of previous MI or coronary artery bypass grafting (CABG), those admitted after more than 6 h of the onset of their symptoms, those having received thrombolytics prior to admission, those with left bundle branch block (LBBB), right bundle branch block (RBBB), intraventricular conduction delay (IVCD) or ventricular rhythms, and those with pacemakers or implantable cardioverter defibrillators were excluded from study. This study was approved by the ethics committee of our University. Informed consent was obtained from all participants. Demographic characteristics, underlying clinical condition, and the duration of time from the onset of symptoms, were recorded for each participant. An ECG using standard method was obtained from each participant at the time of admission. All ECGs were reviewed by two expert cardiologist prior to PPCI. The determination of STEMI was done according to the fourth Universal Definition of Myocardial ischemic ECG criteria. Inverted T wave was defined as a T wave > 1 mm below the isoelectric line in two or more adjacent leads that had maximum ST-segment elevation. In the case of biphasic T wave, it was considered as inverted T wave if T wave was > 1 mm below the isoelectric line in the terminal part of T wave (Fig. 1).

PPCI was conducted by an expert interventional cardiologist and angiographic characteristics including coronary anatomy, the site of the culprit vessel, and thrombolysis in myocardial infarction (TIMI) flow in the IRA were determined. A TIMI flow ≥ 2 (2, 3) was considered as high flow and a TIMI flow < 2 (0–1) as low flow.

### Statistical analysis

Normal distribution of all variables was tested by the Kolmogorov–Smirnov test. Mean and standard deviation of quantitative variables and frequency and percentage of categorical variables were reported. Student’s t-test or Mann–Whitney U were used to compare quantitative variables between the two groups. Chi-square was used for categorical variables. SPSS version 24 (SPSS, Inc., Chicago, Illinois) was used for all analyses. A *p *value < 0.05 was defined as being statistically significant.

## Results

Overlay, 1150 patients with STEMI were studied, of whom 125 patients were excluded due to the following reasons. In 67 patients, there was a positive history of MI or CABG; in 20 patients, LBBB, RBBB or IVCD was present; in 24 patients, duration of chest pain was longer than 6 h; and in 14 patients, streptokinase was administrated prior to hospital admission. Baseline characteristics of the 1025 patients included in the study are described in Table 1. Inverted T wave was seen in 86 (8.4%) patients, among whom 62 patients (72.1%) had anterior and 24 patients (27.9%) had non-anterior STEMI. T wave inversion occurred mostly when IRA vessel for STEMI was the left anterior descending artery. No significant difference was seen in baseline characteristics (including age, sex, and familial history of ischemic heart disease, smoking, time from onset of symptoms, left ventricular ejection fraction, and type of STEMI) of those with inverted T wave and those with positive T waves (Table 1). TIMI flow ≥ 2 was seen in 56.9% of those with inverted T wave while only 9% of patients with positive T wave had a TIMI flow ≥ 2 (*p *value, 0.0001).

**Table 1:** Baseline characteristics of included patients

	Total	Inverted T wave	Positive T wave	*P* value
n (%)	1025	86 (8.4%)	939 (91.6%)	
Age mean ± SD	60.12 ± 12.06	62.11 ± 12.01	60.03 ± 11.09	0.099
*Sex*				
Male	810 (79.0%)	67 (77.9%)	743 (79.1%)	0.790
Female	215 (21.0%)	19 (22.1%)	196 (20.9%)	
Smoking	502 (49.0%)	41 (47.6%)	461 (49.0%)	0.857
*PMH*				
HTN	437 (42.6%)	40 (46.5%)	397 (42.2%)	0.447
DM	234 (22.8%)	20 (23.2%)	214 (22.7%)	0.923
HLP	75 (7.3%)	8 (9.3%)	67 (7.1%)	0.460
Duration of pain (hours) median (min–max)	5 (0.2–6)	4 (0.2–6)	5 (0.8–6)	0.939
LVEF mean ± SD	39.01 ± 8.07	38.09 ± 6.13	39.13 ± 8.09	0.245
*STEMI*				
Anterior	592 (57.8%)	62 (72.1%)	530 (56.4%)	0.008
Non-anterior	433 (42.2%)	24 (27.9%)	409 (43.6%)	
*Infarct related artery*				
LAD	605 (59.0%)	61 (70.9%)	544 (57.9%)	0.059
RCA	303 (29.6%)	17 (19.8%)	286 (30.4%)	
LCX	117 (11.4%)	8 (9.3%)	109 (11.7%)	

*IHD* Ischemic heart disease, *PMH* past medical history, *HTN* hypertension, *DM* diabetes, *HLP* hyperlipidemia, *LVEF* left ventricular ejection fraction, *LAD* left anterior descending artery, *RCA* right coronary artery, *LCX* left circumflex artery

**Fig. 2:**
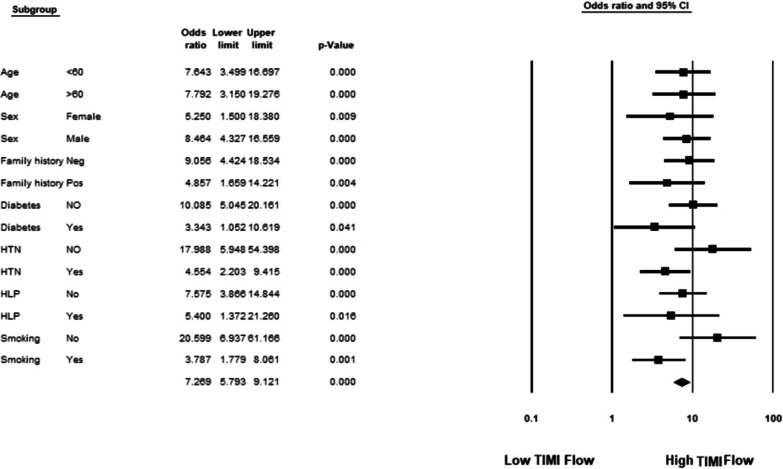
Association between TIMI flow and T wave inversion in patients with anterior ST-elevation MI analysed in different subgroups

**Fig. 3:**
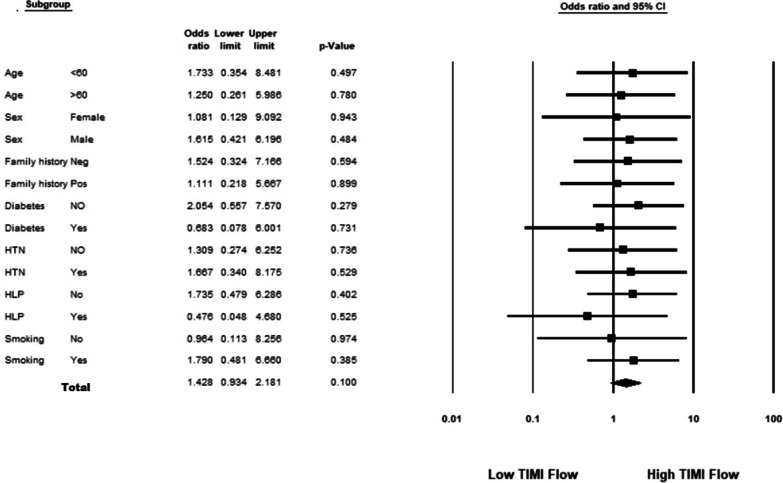
Association between TIMI flow and T wave inversion in patients with non-anterior ST-elevation MI analysed in different subgroups

Anterior STEMI was seen in 592 patients (57.8%), of which 62 patients (10.4%) had concomitant on admission inverted T wave. In patients with anterior STEMI and a negative T wave TIMI flow was significantly higher (χ^2^ [1, N = 592], 203.41; *p *value, < 0.001); however, in non-anterior STEMI group, an on admission negative T in leads with ST elevation was not associated with high TIMI flow (χ^2^ [1, N = 433], 0.46; *p *value, 0.496; Table 2). The same associations remained in sub-group analysis in both anterior (Fig. 2) and non-anterior STMI patients (Fig. 3). The sensitivity, specificity, positive, and negative predictive values of inverted T wave for diagnosis of high TMI flow were 55.56, 96.67, 72.58, and 93.21%, respectively.

**Table 2:** The relation between T wave inversion in anterior and non-anterior STEMI with TIMI flow

Individuals	TIMI flow		
	High	Low	*P* value
*Anterior STEMI*			
Inverted T wave	45 (72.6%)	17 (27.4%)	< 0.001
Positive T wave	36 (6.7%)	494 (93.3%)	
*Non-anterior STEMI*			
Inverted T wave	4 (16.6%)	20 (83.4%)	0.496
Positive T wave	49 (11.9%)	360 (88.1%)

## Discussion

We evaluated the applicability of T wave inversion for predicting IRA patency in a prospective large-scale study. The results pointed to a significant relationship between on admission T wave inversion and patency of the culprit vessel in acute anterior STEMI.

Current guidelines of AHA or ESC advises PPCI or thrombolytics for all STEMI patients admitted within the initial hours of the onset of symptoms. However, as our results showed, a significant percent of patients with STEMI had spontaneous reperfusion of IRA. The incidence of SR in STEMI has been variedly reported by previous studies in a range of 7–57% [2–7]. This discrepancy could be due to different definitions of SR or the timing of assessments [13]. In our study, we used angiography for the determination of SR, which was suggested as the most reliable method in previous research [2–7].

By means of a better identification of patients with SR, a significant proportion of patients previously indicated for PPCI can be considered for conservative treatments [4, 5, 8]. In order to design an appropriate reperfusion protocol that considers the possibility of SR, sensitive and specific diagnostic tools are necessary to detect the presence or absence of SR prior to PPCI. Due to the pressure of time for rapid revascularization of patients with STEMI, imaging or other laboratory tests may not be beneficial. However, ECG as an available, readily applicable, low-cost, and non-invasive tool can be considered on these occasions. Our results support the applicability of T wave inversion in ECG as a marker of SR. Consistent with our findings, Hira et al. suggested that negative T wave upon the presentation of ECG could be a sign of spontaneous thrombolysis in patients with anterior STEMI [11]. Nakajima et al. demonstrated that the amplitude of negative T wave within 48 h of MI was inversely correlated with the extent of hypokinetic area of the heart; this predictive power was appreciated in both anterior and inferior STEMIs [14]. However, there was no relationship between T wave inversion and patency rate of non-anterior STEMI patients in our study. Similarly, this association in non-STEMI was not confirmed in the study by Hira et al. [11]. The reported positive and negative predictive values by the study of Hira et al. were similar to those emerging in our study. However, they reported a higher sensitivity and relatively lower specificity, which could be due to retrospective design and relatively small sample size in that study. The sensitivity and specificity reported by Alsaab et al. were 43.8 and 89.2%, respectively [12]. Studies on the effectiveness of PPCI have also reported T wave inversion as an important element for successful reperfusion. Hirota et al. indicated that negative T wave develops within only 0.5–5 h of successful reperfusion [15]. Moreover, non-inverted T wave 24 h after anterior STEMI predicted a LVEF less than 40%, by 90% sensitivity and 75% specificity [16]. The cornerstone of higher IRA patency in patients with negative T wave and anterior STEMI is unknown. More collateral circulation in LAD and RCA territories was shown in Wang et al. [17] study. Better perfusion through collateral arteries and subsequently better endothelial function could maintain blood flow after spontaneous reperfusion.

Considering ischemic and bleeding complications of PPCI [18] and given the fact that PPCI is not available in some medical centers and that fibrinolytic therapy can be life-threatening in patients who are at risk of bleeding, it seems that fibrinolytic therapy in patients with acute anterior STEMI and early T wave inversion upon ECG presentation (who have ST elevation in on admission ECG but negative T wave present in leads with ST elevation, too), especially in those with concomitant chest pain reduction, may not be an appropriate therapeutic strategy.

Our study suffered from some inevitable limitations. Due to the large number of patients included in the study, PPCI and assessment of TIMI flow could not be performed by a single cardiologist to reduce possible discrepancies. Having said that, we did make attempts to follow the standard method for all patients. Moreover, due to the prospective design of the study, the cardiologist that performed PPCI was not blinded to patients’ ECG.

TIMI flow is considered a strong predictor of long term outcome of patients with STEMI [19]. However, future studies are warranted to evaluate the long term outcome of STEMI patients presenting with positive/inverted T wave.

## Conclusion

In on-admission ECG of patients with anterior STEMI, concomitant inverted T wave in leads with ST elevation could be a good marker of spontaneous reperfusion of infarct related artery.

## Data Availability

All data and material collected during this study are available from the corresponding author upon reasonable request.

## Abbreviations

ECG: Electrocardiography
LVEF: Left ventricular ejection fraction
LAD: Left anterior descending artery
RCA: Right coronary artery
STEMI: ST elevation myocardial infraction
PPCI: Primary percutaneous coronary intervention
TIMI: Thrombolysis in myocardial infarction
MI: Myocardial infraction
CABG: Coronary artery bypass grafting
LBBB: Left bundle branch block
RBBB: Right bundle branch block
IVCD: Intraventricular conduction delay
